# Assessment of the National Test Strategy on the Development of the COVID-19 Pandemic in Denmark

**DOI:** 10.3390/epidemiologia2040037

**Published:** 2021-11-05

**Authors:** Peter Kamp Busk, Thomas Birk Kristiansen, Allan Engsig-Karup

**Affiliations:** 1 Department of Science and Environment, Roskilde University, 4000 Roskilde, Denmark; 2 Ishøj Centrets Læger, 2635 Ishøj, Denmark; thomasbirk@dadlnet.dk; 3 Department of Applied Mathematics and Computer Science, Technical University of Denmark, DK-2880 Kgs. Lyngby, Denmark; apek@dtu.dk

**Keywords:** COVID-19, mass testing, rt-PCR, antigen test, hospitalization, vaccination

## Abstract

During the COVID-19 pandemic, Denmark has pursued a mass testing strategy culminating in the testing of 12.167 individuals per 100,000 inhabitants per day during the spring of 2021. The strategy included free access to COVID-19 testing, and since 2021, compulsory documentation for negative tests or vaccination has been required for access to workplace, educational institutions, restaurants, and many other places. Testing and subsequent isolation if testing was positive were voluntary. The present study provides an analysis of whether testing frequency in Denmark showed any correlation to hospitalizations throughout the relevant stages of the pandemic. Mass testing was found not to correlate significantly with the number of hospitalizations during the pandemic. Interestingly, during the highest level of testing in spring 2021 the fraction of positive tests increased slightly; thus, the Danish mass testing strategy, at its best, failed to reduce the prevalence of COVID-19. Furthermore, the relationship between positives in antigen testing and in rt-PCR testing indicated that many patients were not tested early in their infection when the risk of transmission was at the highest. In conclusion, the Danish mass testing strategy for COVID-19 does not appear to have a detectable correlation to the number of hospitalizations due to COVID-19.

## 1. Introduction

Early in the coronavirus disease (COVID-19) pandemic, mass testing for severe acute respiratory syndrome coronavirus 2 (SARS-CoV-2) was suggested as a means to find and isolate infected individuals [1]. The assumption was that by systematically testing every citizen once a week, it would be possible to bring the pandemic to a halt in a span of a few months. Arguments against mass testing included concerns about false positives at low incidence, the high cost of mass testing, and concerns about the adherence to self-isolation of quarantined individuals [2].

From the spring of 2020, Denmark emphasized mass testing of the population as a means to combat the pandemic. The first case of SARS-CoV-2 infection in Denmark was diagnosed on 27 February 2020 [3]. Within a month, Danish authorities decided to introduce large-scale COVID-19 diagnostic testing [4]. In a short time period, the capacity for reverse-transcription polymerase chain reaction (rt-PCR) testing for SARS-CoV-2 was expanded such that every citizen had the right to be tested for free without prior medical evaluation from the end of May 2020 [5]. Thus, in December 2020, COVID-19 testing in Denmark reached 2900 tests per day per 100,000 inhabitants [6]. By implementation of rapid antigen tests for mass screening purposes, the Danish test capacity was further increased and peaked at 12,167 COVID-19 tests per day per 100,000 inhabitants in May 2021. As part of a reopening plan for Denmark in March 2021, a negative COVID-19 test or completion of COVID-19 vaccination became compulsory for attending education, restaurants, fitness centers, etc. [7]. Moreover, children in elementary schools were strongly urged to take biweekly COVID-19 tests.

In comparison, in late 2020, Slovakia tested the entire population for COVID-19 twice in one week through mandatory testing. The short-term effect of this systematic antigen testing was a reduction in the incidence of the disease by 70% compared to no testing [8], although the effect on hospitalizations was limited [9]. The same approach was repeated two weeks later in regions with high incidence [10]. In contrast to Slovakia, the Danish strategy was based mostly on voluntary testing but included compulsory testing for participation in educational and recreational activity and for admittance to workplaces. As testing in Denmark was unsystematic, there was a large difference in the testing of each citizen. The number of tests since the outbreak of the pandemic until 1 July 2021 corresponded to an average of 11 tests per citizen; nevertheless, approximately 900,000 thousand persons, comprising 10% of the population above five years of age, had not been tested even once for COVID-19 [11]. Hence, the Danish test strategy was different from the systematic weekly testing suggested at the beginning of the pandemic [1] and from the tests carried out in Slovakia [8,9,10].

In a recent study, the effect of the Danish test strategy was assessed by comparing actual test data to an extrapolation of the expected number of positive tests if Denmark had followed a low testing strategy similarly to Norway and Sweden and to an estimation of the total number of contaminated persons in the country [12]. These calculations suggest that testing at the scale implemented in Denmark reduces contact number by 25%.

In the present study, we assessed the observable impact of extensive COVID-19 testing on the Danish population primarily by examining the correlation between the number of tests and the number of hospitalizations in meaningful periods of the COVID-19 pandemic. This is performed by calculating the Pearson correlation coefficient between the number of tests performed and the number of hospitalizations. The effect on hospitalizations of testing and quarantine of infected individuals is expected to be delayed. However, no negative correlation between the number of tests and hospitalizations were found for a delay of 7, 14 or 21 days.

## 2. Materials and Methods

Data for number of tests, number of positives, and hospitalizations were downloaded from Statens Serum Institut’s daily report on 24 June 2021 [11]. Data for vaccinations were downloaded on 5 July 2021 [13]. All data are available in Supplementary Materials.

Correlations were calculated as Pearson correlation coefficients between the data sets [14]:

(1) $$r_{xy}=\frac{\sum_{i}\left(x_{i}-\bar{x}\right)\left(y_{i}-\bar{y}\right)}{\sqrt{\sum_{i}{\left(x_{i}-\bar{x}\right)}^{2}}\sqrt{\sum_{i}{\left(y_{i}-\bar{y}\right)}^{2}}}$$

where the sample size of each data sets is of size *n* and ordered in a set of indiced pairs {$\left(x_{i},y_{i}\right)$, for *i* = 1,…, *n*}. The sample means of each data set were calculated from the following.

(2) $$\bar{x}=\frac{1}{n}\sum_{i}x_{i},\;\bar{y}=\frac{1}{n}\sum_{i}y_{i}$$

The Pearson correlation coefficient varies in the range −1–1 and measures in simple terms the strength and direction of a linear relationship between variables. To evaluate the statistical significance of the correlation and whether it is valid for the entire population, *p*-values are calculated based on a Student’s *T*-test. The *p*-value is the probability that the correlation coefficients determined were in fact zero and can be used to either accept the *null hypothesis* that $r_{xy}=0$ or to reject it $r_{xy}\neq 0$. Hence, the *p*-value indicates whether correlation between the data sets occurred by chance. A low *p*-value is better and is considered statistically significant if smaller than 0.05 (α value of 5%).

All relevant ethical guidelines have been followed.

## 3. Results

The number of COVID-19 positives detected inherently correlates to the number of tests performed; however, the number COVID-19 positives is also influenced by whether right persons of interest are tested at the right time. In contrast, hospitalization depends on the patient’s symptoms; thus, it is a more suitable measure of the development of the pandemic. Hence, if the mass testing strategy has an effect on reducing COVID-19 infections, the number of hospitalizations is expected to show a negative correlation to the number of tests.

### 3.1. Testing Stages

**Figure 1:**
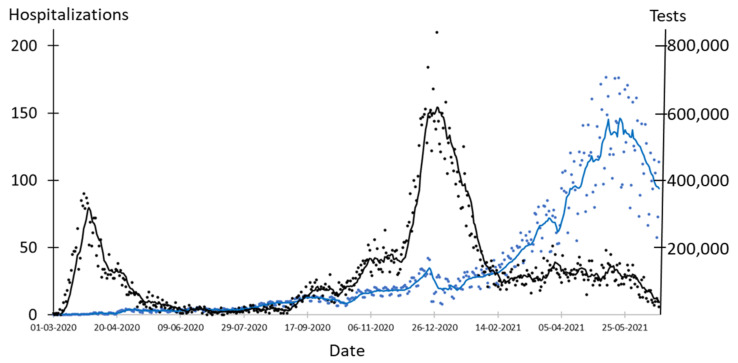
Number of COVID-19 tests and hospitalizations in Denmark during the pandemic. Blue dots: number of tests. Blue line: seven days average of number of tests. Black dots: hospitalizations. Black line: seven days average of hospitalizations.

Figure 1 shows the number of COVID-19 tests and hospitalizations since the first patient diagnosed with COVID-19 in Denmark was hospitalized on 1 March 2020 until 21 June 2021. Mass testing can be divided into stages 0–3.

- Stage 0 (1 March 2020 to 26 May 2020): The test capacity was set up, and the number of daily tests increased from 49 to 15,980. This period includes the lock down period 12 March 2020–26 May 2020 [15,16].
- Stage 1 (27 May 2020 to 6 December 2020): Danish society was open with relatively few changes in restrictions, although compulsory facemasks in public transport were introduced on 22 August and in all indoor facilities with public assess on 29 October [17,18]. During this stage, testing increased from 14,781 tests per day to 69,215 tests per day. Self-reported concerns about the pandemic were at a high level and increasing support to measures implemented by authorities to keep distance and lower the number of contacts was reported [19,20].
- Stage 2 (11 December 2020 to 28 February 2021): A winter lockdown with severe restrictions was implemented [21]. Hospitalizations peaked at 210 on 28 December 2020 and testing increased from 102,431 tests per day to 143,001 tests per day. Vaccinations started on 27 December and reached 7.3% of the population; most comprised elderly on 28 February [6,22]. Awareness of social distancing and of reducing the number of contacts was high and increased during this period [20]. The population was concerned, and the support for following advice from authorities was high [19].
- Stage 3 (6 April 2021–21 June 2021): Schools, other educational institutions, and small businesses such as hairdressers, tattoo hops, massage parlors, and others were reopened [23]. Malls were reopened on 21 April and many indoor facilities such as restaurants and museums opened on 6 May for guests that had been vaccinated or had a negative COVID-19 test not older than 72 h [24]. The requirement for facemasks was abandoned on 14 June [25]. This period includes the most extensive level of mass testing with an average of 466,861 tests per day corresponding to 8049 tests per day per 100,000 persons. By the end of May, all citizens above 65 years had been offered a COVID-19 vaccine, and 37.4% of the population had received the first dose [3,15]. On 21 June 2021, 53% of the population had received the first dose. Awareness of social distancing and of reducing the number of contacts was low and decreased during this period [20]. Adherence to self-isolation was low. The population was not that worried and reported decreasing will to follow advice from authorities [19].

### 3.2. Effect of Testing on Hospitalizations

**Figure 2:**
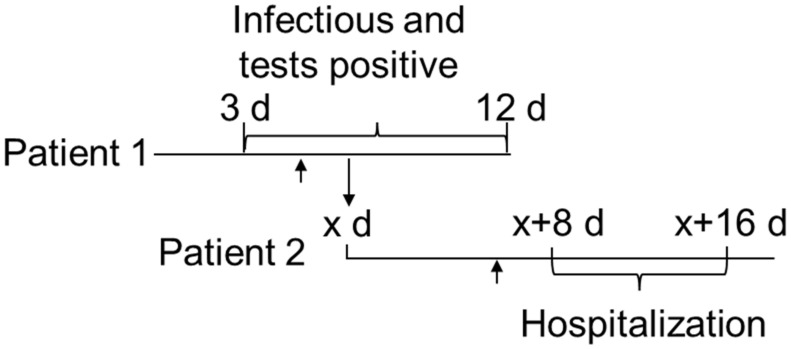
Overview of timing of infectivity, positive test, and hospitalizations for two patients. Patient 1 is infected on day 0 and can infect others from day 3–12 (3 d–12 d). Patient 2 is infected by patient 1 on day x and risks hospitalization between day x + 8 and x + 16. The arrows indicate expected onset of symptoms on day 5 after infection.

The incubation period for COVID-19 until a patient is infectious and tested positive in an rt-PCR or an antigen test is 2.5–7 days [26,27,28,29]. Without testing and quarantine, the index patient (patient 1, Figure 2) can infect the next patient (patient 2, Figure 2). After an additional 8–16 days, patient 2 will be hospitalized [30]. Thus, there is a considerable delay from the infectious contact between patients 1 and 2 at day x to the hospitalization of patient 2 on day x + 8 to x + 16 (Figure 2). As the window of passing COVID-19 to others is roughly 3–12 days, post infection x can vary from 3 to 12 days, implying that hospitalization of patient 2 occurs 11 to 28 days after the infection of patient 1.

Any negative impact of a positive test on the hospitalization of patient 2 presupposes that patient 1 was tested and found positive three to ten days prior (day 3-x to 10-x) and was quarantined to avoid infection of patient 2. Hence, if patient 1 was quarantined due to a positive test, the number of hospitalizations 11–28 days later is expected to decrease. This should result in a negative correlation between the number of tests and the number of hospitalizations.

However, no clear negative correlation was observed during the period of mass testing in Denmark (27 May 2020–21 June 2021) or during each of the mass testing stages 1, 2, and 3 (Table 1). The lack of correlation was independent of the expected delay time (7, 14, or 21 days) between test and hospitalization.

Self-reported data suggest that the population was highly aware of keeping distance and reducing the number of contacts during stages 1 and 2 and that the awareness was increasing during these stages [19,20]. This behavior works in favor of reduced hospitalization and, thus, in favor of a negative correlation between the increasing number of tests and hospitalization. However, no negative correlation was found (Table 1). The weak correlations found for stages 2 and 3 were not significant (*p* > 0.05). In stage 3, the willingness to follow advice, to keep distance, and to reduce the number of contacts was lower and decreased [19,20]. Although the willingness to follow advice from the authorities was highest during stage 2 [19], self-reported adherence to quarantine after a positive COVID-19 test was only 50% in December 2020 [31]. Thus, breaching quarantine was a major issue during all stages.

In stage 0 before mass testing was initiated, there was a strong negative correlation between the number of tests performed and hospitalizations (Table 1). However, it is not clear whether the decrease in the number of hospitalizations during this stage was due to low-level testing or caused by the lockdown, seasonal variations, or other factors.

Surprisingly, there is a strong, positive correlation (*p* < 0.001) between the number of tests and hospitalizations for stage 1 (Table 1). Clearly, testing per se does not increase contamination with COVID-19; thus, any correlation must be due to other factors, such as increased awareness of disease, resulting in an increased motivation for voluntary testing.

### 3.3. Effect of Testing on Percentage of Positive Tests

In a voluntary mass testing regime employed in Denmark, the percentage of COVID-19 tests that are positive depends on the prevalence of COVID-19 in the population, the number of persons tested, and the ability for testing individuals of relevance. When the number of tests is relatively constant over a shorter period, it is reasonable to assume that the percentage of positive tests reflects the prevalence of COVID-19 in the population.

**Figure 3:**
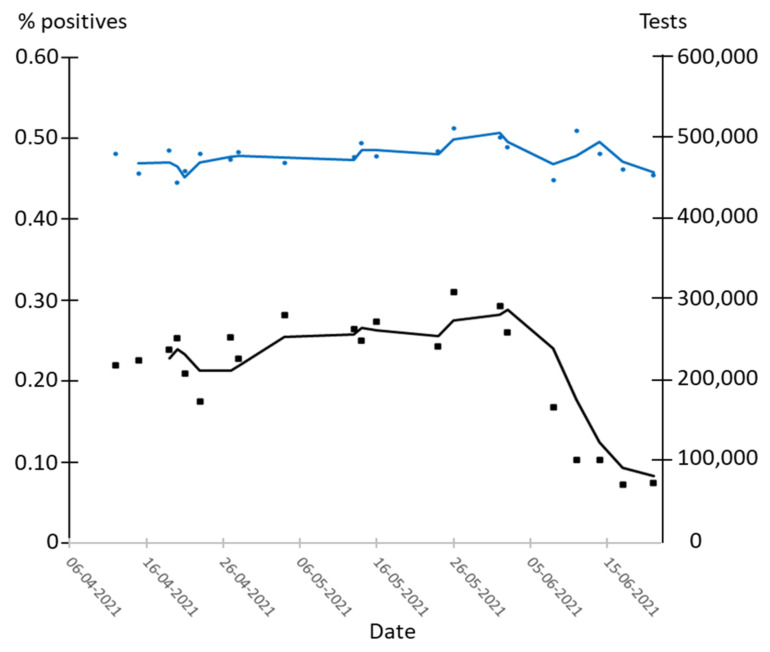
Percentage of positive tests at approximately the same number of daily tests during April–June 2021. Blue dots: number of tests. Blue line: three days average of number of tests. Black dots: % positive tests. Black line: three days average of % positive tests.

In order to further assess the effect of mass testing in Denmark during stage 3, the positive percentage was calculated for all days in the period 6 April to 21 June 2021, where the total number of daily tests was relatively constant within 444,500 to 512,313 (Figure 3). Although the number of tests was almost constant (477,215 tests ± 7%), the percentage of positive tests increased slightly from 0.22 on 12 April to 0.31 on 26 May and then decreased rapidly to 0.07 on 17 June (Figure 3). In the week before 12 April, the average percentage of positive tests was 0.24 on an average of 375,000 tests per day, whereas in the week after 26 May, the average percentage of positive tests was 0.27 on an average of more than 525,000 tests per day.

Testing was expected to reduce the number of patients testing positive within x + 3 days to x + 12 days (Figure 2). Hence, for x between day 3 and 10, mass testing should reduce the percentage of positive tests within 6–22 days. However, the increase in percentage of positive tests over the 44 days of mass testing between 12 April and 26 May does not indicate that the Danish strategy of mass testing effectively reduced the prevalence of COVID-19. This could be due to low support for recommended measures such as quarantine at this stage [19]. Even at high support for the recommended measures, only 50% self-reported adherence to quarantine [31]; thus, in stage 3 self-isolation upon a positive COVID-19 test was likely low.

The decrease in percentage positives below 0.22 after 2 June (Figure 3) came too late to be a direct effect of mass testing and may be a seasonal effect or an effect of vaccination of more than half of the population [6,22,32].

### 3.4. Ratio between Positive Antigen Tests and Rt-PCR Tests

Sensitivity of the antigen tests relative to the rt-PCR test is on average 78% during the first week after symptom onset decreasing to 51% during the second week [24]. For patients later in the second week after onset of symptoms, sensitivity drops even further. Hence, the percentage of rt-PCR positive tests that was also positive in an antigen test can be used to measure whether tests are performed in week 1 of the infection cycle when patients are likely to infect others [26]. Curiously, from 1 February to 20 June 2021, the antigen tests only detected COVID-19 in 48% of the samples that were positive in rt-PCR. This is much lower than the expected 78% if the patients were tested in week 1 after symptom onset [33]. The relation between positive antigen tests and positive rt-PCR tests for the same patient suggests that many patients detected positive by rt-PCR may be from week 2 or later after infection where they were no longer highly infectious, thus, undermining the voluntary strategy of mass testing in Denmark.

To be effective for reducing COVID-19 prevalence and hospitalizations, mass testing should detect infected patients before symptom onset or early in week 1 after symptoms developed; otherwise, transmission cannot be halted.

**Figure 4:**
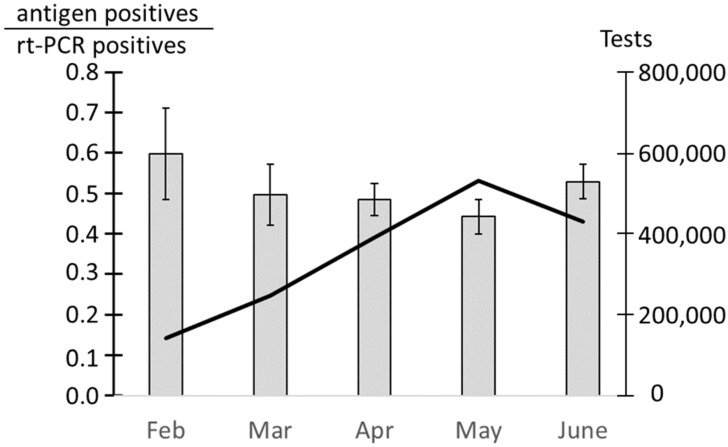
Variation in the number of positive rt-PCR samples that are positive in an antigen test in Spring 2021. Grey bars: fraction of rt-PCR positive test that are also positive in an antigen test ± standard deviation. Solid line: total number of tests performed.

Antigen tests are most effective in the beginning of the infection; hence, as testing increased in Denmark during spring 2021, the percentage of rt-PCR positives, which were also detected by antigen tests, was expected to increase. However, from February 2021 to May 2021, when testing peaked, the percent of rt-PCR positives that were also detected by antigen tests decreased from 60% to 44% before increasing to 53% in the first three weeks of June (Figure 4).

The average ratio of antigen positives to rt-PCR positives on the same patients for February to March was 48%, whereas testing of the target group of patients in week 1 of the COVID-19 infection should yield a ratio of 80% or more [33]. Assuming a sensitivity of the antigen test of 0.8 in week 1 and 0.3 in weeks 2 and later, the observed ratio between antigen positives and rt-PCR positives suggests that only 36% of the tested persons are in the target group for testing (0.48 = (0.8 × w1 + 0.3 × w2)/(w1 + w2)), where w1 is the rt-PCR positives in week 1, and w2 is rt-PCR the positives in week 2 and w1 + w2 = 100. This should be regarded as a lower limit, as it depends on an assumption that antigen tests and rt-PCR tests were performed at the same time. This was often not the case. The number of COVID-19 positives is based on antigen and rt-PCR tests performed within 48 h. However, if the rt-PCR test was performed later than the antigen test, patients in the first week of infection may have had time to increase their viral load and, thus, be more likely to test positive. This will skew the ratio towards lower numbers. Nevertheless, even a sensitivity of the antigen tests as low as 0.6 during the first week of infection implies that 40% of the rt-PCR positives are outside the target group for testing. Thus, a high level of irrelevant tests was performed.

### 3.5. Vaccination and Hospitalizations Correlated

**Figure 5:**
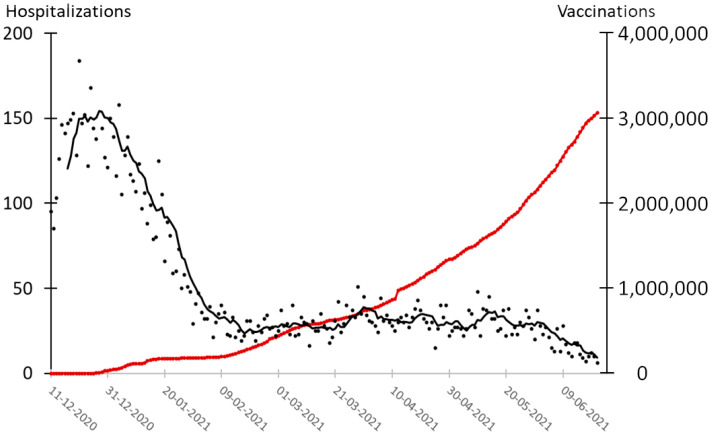
Number of hospitalizations and vaccinations in Denmark during the pandemic. Black dots: hospitalizations. Black line: seven days average of number of hospitalizations. Red line: accumulated number of vaccinations.

In contrast to what was observed for testing, there was a strong negative correlation of −0.50 (*p* < 0.001) between the accumulated number of vaccinations and hospitalizations 14 days later for the period when vaccination was initiated on 27 December 2020 to 21 June 2021 (Figure 5). For stage 3 (6 April–21 June 2021), the correlation was −0.66 (*p* < 0.001), which agrees with a significant effect of vaccinations on the number of hospitalizations.

## 4. Discussion

The present investigation does not indicate that mass testing in the Danish context reduced the prevalence of COVID-19 effectively, as the number of tests and hospitalizations do not correlate. In particular, the lack of correlation during stage 3 from 6 April to 21 June 2021, when an average of more than 8000 in 100,000 persons were tested per day, does not support mass testing at the scale implemented in Denmark as a tool to reduce prevalence of COVID-19.

Testing at this magnitude is sufficient for testing the entire population in less than two weeks in a scheme resembling the form of mass testing that took place in Slovakia in the fall of 2020 [8]. However, systematic testing in Slovakia reduced COVID-19 prevalence by 70% compared to unmitigated growth and by 58% in absolute numbers within a week of testing [8].

Although it cannot be excluded that mass testing in Denmark during spring of 2021 impaired an increase in the number of hospitalizations and the percentage of positive tests, this study provides no evidence to support this hypothesis. This is in contrast to vaccinations against COVID-19 where the present analysis finds a correlation between the accumulated number of vaccinations and hospitalizations, as expected [34].

If the Danish test strategy had demonstrated a similar impact, as the first round of testing did in Slovakia in the fall of 2020, it would have shown up as a significant negative correlation between testing and hospitalizations and further as a decrease in the percentage of positive tests. However, there was no significant rapid decrease in the percentage of positive tests when comparing the percentage of positives on days with similar numbers of tests during stage 3.

It is reasonable to attribute the decrease in the percentage of positives towards the end of stage 3 to an improvement in the weather conditions and vaccination of more than one-third of the population by the end of May. Both summer weather conditions and vaccinations reduced the prevalence of COVID-19 [32,35].

In Slovakia, it was observed that the effect of massive testing was limited to regions with high COVID-19 prevalence, whereas testing did not seem to have an effect in regions with low prevalence [8]. This is consistent with the assumption that high prevalence, which was not the case in Denmark during spring 2021, is a prerequisite for an impact of mass testing [2,36].

Another difference when comparing Slovakia to Denmark is that Denmark did not perform systematic testing of all citizens. Consequently, testing was unequally distributed, as 10% of the population above five years of age was not at all tested for COVID-19 [11], and test frequency for the tested varied. Unequal testing implies that some people were tested often, whereas others were tested sporadically or not at all.

An analysis of mass testing in the canton of Grisons in Switzerland found that testing was ineffective when the tested individuals were in close contact with untested individuals [37]. Under these conditions testing was futile as the tested individuals were easily contaminated by untested individuals.

Along the same lines, the second round of mass testing in Slovakia decreased COVID-19 infections by 30% but the effect disappeared within three weeks after testing [10]. Moreover, hospitalizations plateaued a few weeks after mass testing but then increased [9]. It is likely that the effect of mass testing in Slovakia was reversed by contamination from sources such as undetected false negative cases and interaction with contaminated individuals from other districts or abroad.

In Denmark, daily testing of 10% of the population across the country implied that these individuals were exposed to COVID-19 from up to 90% of the population that was not tested. Focused testing on workplaces, schools, restaurants, etc., [24] counteracted the mixing of tested and untested individuals to some extent, but it did not prevent cross-contamination in public transport, supermarkets, and other unrestricted public spaces. Moreover, in December 2020, it was found that only half of the individuals that tested positive for COVID-19 in Denmark self-reported that they complied with quarantine rules, although this was actually the period where most people followed advice from the authorities [20,31]. This breach of quarantine is a factor contributing to the insignificant impact of mass testing in Denmark. Thus, concern about adherence to self-isolation, as expressed by Gill and Gray, is important [2].

The uneven distribution of testing in Denmark was likely to be a factor in lowering the ratio between rt-PCR positives and antigen test positives. A plausible explanation for this low ratio was that a large fraction of the testing was not performed on the target group of infectious persons but rather on people with late-stage COVID-19, where the risk of transmission was low or absent [26]. In a strict sense, rt-PCR positives with late-stage COVID-19 can be regarded as false positives in a screening program designed to find highly infectious individuals.

The estimation that only 36% of the rt-PCR positives were in the target group of infectious persons implies that 64% of the rt-PCR positives were outside the target group. This number should be regarded as an upper limit as it depends on several assumptions about the testing. Nevertheless, the results suggest that a large fraction of rt-PCR positives were outside the target group for mass testing as these persons were likely to have late-stage COVID-19 with low or absent risk of infecting others [26].

It would be interesting to confirm this possibility by a detailed follow up study of the COVID-19 development in many individuals tested positive for COVID-19 in the Danish mass testing program.

An implication of a large fraction of COVID-19 infections passing undetected until late-stages is that mass testing, as carried out, has not been efficient in finding the patients that are infectious.

From a theoretical point of view, it is logical to assume that the detection of COVID-19 positives by mass testing to place infected individuals in quarantine could break the chains of infection and reduces prevalence of the disease. A bottom-up calculation reached the conclusion that the Danish mass testing program in spring 2021 reduced the prevalence of COVID-19 by removing 25% of the infected patients every day [12]. This conclusion was based on an extrapolation of the expected number of positive tests if Denmark had followed a low-testing strategy such as its neighboring countries: Norway and Sweden [12]. The extrapolation assumed that if Denmark had performed 30,000 tests per day, approximately 200 COVID-19 positive patients would have been found.

The authors express their reservations as to whether the extrapolation is valid under the conditions in the spring of 2021. Indeed, the actual testing carried out in Norway in the same period detected on average 550 COVID-19 positives per day, although only 16,000–27,000 tests were performed [6]. Hence, the assumption that 30,000 tests per day would only detect 200 positives [12] seems to be a large underestimation.

Low-level testing in Denmark would have identified more than 550 COVID-19 cases per day in spring 2021 provided that the Danish health authorities are as efficient as the Norwegian authorities were on COVID-19 testing. If the bottom-up calculations [12] are adjusted to this notion, mass testing during spring 2021 detected less than 10% of the infected persons each day.

In this context it is interesting that there was a correlation between the number of tests and reduction in hospitalizations in Denmark during spring 2020 when testing was performed at a low level. This correlation raises the possibility that low-level testing of symptomatic patients and close contacts has positive impacts on the control of the pandemic but that further increase in testing is futile. However, the observed negative correlation should be interpreted carefully as other measures such as an increased awareness of the pandemic, lockdown, and weather conditions are likely to have had a high impact on the number of hospitalizations in the spring of 2020 [32,38].

It is reasonable to assume that low impact of mass testing (10% decrease in prevalence) would not be detectable when comparing the number of tests to hospitalizations. Hence, a calculation based on the assumption that the Danish health authorities are as efficient as the Norwegian authorities agrees with the observations in the present study that mass testing does not have a measurable impact on COVID-19 prevalence in Denmark.

The question remains: why did mass testing not impact COVID-19 prevalence? One reason could be, as described in the present study, that testing for a large part was performed outside the target group of early stage infectious persons. This could be attributed to the unsystematic approach to testing utilized in Denmark. Moreover, recently tested individuals were in contact with the untested part of the population, and there was low compliance with quarantine requirements [2,31].

Lieberman, Lieberman, and Bourassa suggest that testing induces unsafe behavior resulting in increased transmission of COVID-19 thereby outweighing the positive effects of testing [39]. According to this possibility, individuals that tested negative ignore the considerable risk of false negatives of 0.20 and 0.15, respectively, for the antigen and the rt-PCR tests [33,40]. Instead, the negative test result increases risk behavior. This is a general phenomenon in diagnostics described as preventive misconception [41]. A simple thought experiment can demonstrate how elevated risk behavior can outweigh mass testing: The risk of transmission of COVID-19 from one person to another depends on the third power of the distance between the two. If mass testing removes 25% of COVID-19 infected patients every day as suggested [12], it only takes a 10% decrease in the average distance between the rest of the citizens to offset the effect of airborne transmission. Thus, only a small increase in risk behavior regarding distance and other COVID-19-related precautions can neutralize the effect of mass testing. Preventive misconception combined with false negatives could also be an import factor driving the reversal of positive effects of COVID-19 mass testing in Slovakia [9,10].

Neither Denmark nor Slovakia followed the early suggestion of strictly systematic weekly COVID-19 testing of the entire population [1]. However, the Danish and Slovak cases suggest that positive effects of mass testing could be outweighed by false negative rates (which could be as high as 0.15 even for the best tests [40]), breaching quarantine, and preventive misconception. Moreover, analysis of the testing in canton Grisons demonstrated that mixing the tested part of the population with the untested population neutralizes the effect of mass testing [37]. Means to counteract these factors and the considerable costs of testing should be carefully considered before mass testing is implemented [2]. Interestingly, post lock-down mass testing by rt-PCR in Wuhan from 14 May 2020 was performed on 9.9 million individuals (93% of the population) in only 19 days followed by compulsory and controlled quarantine [42]. In general, Chinese provinces have followed a strategy characterized by concentrated and comprehensive mass testing followed by compulsory quarantine, e.g., in a centralized, controlled facility [43]. For example, after detecting three COVID-19 cases in Qingdao, a complete lockdown and mass testing of 10.9 million individuals in only 5 days followed by compulsory quarantine was able to contain the outbreak [44]. The fast and comprehensive testing prevents mixing tested individuals with untested individuals. Moreover, compulsory and supervised quarantine eliminates the risk of relying on voluntary self-isolation. In societies where there is no political and societal support for such extreme measures, mass testing does not appear to be an efficient mean for combating the COVID-19 pandemic.

## 5. Conclusions

In Denmark, the implementation of mass testing was not found to correlate with the number of hospitalizations due to COVID-19. Furthermore, the ramp up of mass testing during the spring of 2021 does not appear to have lowered the number of positive tests at comparable test numbers. On the contrary, the number of positive tests increased slightly between 12 April and 26 May 2021, thus underpinning that mass testing did not reduce the prevalence of COVID-19 at best.

Likely explanations for the apparent lack of impact of mass testing in a Danish context is that individuals with a negative test were in contact with the untested part of the population, many were tested too late, sporadic testing caused much irrelevant testing, the testing may increase the risk behavior of tested persons, and individuals with a positive test breached the quarantine requirement.

## Figures and Tables

**Table 1:** Correlation between number of tests and hospitalizations. Statistical significance is indicated with *p*-values for the correlations in parenthesis.

Stage	Dates	Average Daily Tests per 100,000 Inhabitants	Average Daily Hospitalizations	Correlation 7 Days Delay	Correlation 14 Days Delay	Correlation 21 Days Delay
All	27 May 2020–21 June 2021	2722	34	0.02 (0.66)	0.00 (0.99)	−0.02 (0.74)
0	01 March 2020–26 May 2020	114	28	−0.61 (0.00)	−0.71 (0.00)	−0.66 (0.00)
1	27 May 2020–06 December 2020	652	16	0.87 (0.00)	0.83 (0.00)	0.81 (0.00)
2	11 December 2020–28 February 2021	1949	83	−0.22 (0.06)	−0.06 (0.65)	0.07 (0.61)
3	06 April 2021–21 June 2021	8049	27	0.16 (0.19)	−0.06 (0.63)	−0.24 (0.07)

## Data Availability

Data were obtained from https://COVID19.ssi.dk/overvagningsdata/download-fil-med-overvaagningdata (accessed on 24 June 2021) and https://COVID19.ssi.dk/overvagningsdata/download-fil-med-vaccinationsdata (accessed on 5 July 2021) and can be found in Supplementary Materials.

## Supplementary Materials

The following are available online at https://www.mdpi.com/article/10.3390/epidemiologia2040037/s1, Table S1: Danish testing results, Table S2: Hospitalizations, Table S3: Double testing, Table S4: Vaccinations.
